# Confounding with familial determinants affects the association between mode of delivery and childhood asthma medication – a national cohort study

**DOI:** 10.1186/1710-1492-9-14

**Published:** 2013-04-16

**Authors:** Lennart Bråbäck, Cecilia Ekéus, Adrian J Lowe, Anders Hjern

**Affiliations:** 1 Occupational & Environmental Medicine, Department of Public Health and Clinical Medicine, Umeå University, Umeå, Sweden; 2 Department of Research and Development, Västernorrland County Council, Sundsvall, Sweden; 3 Department of Women’s and Children’s Health, Division of Reproductive and Perinatal Health, Karolinska Institutet, Stockholm, Sweden; 4 Murdoch Childrens Research Institute, Melbourne, Australia; 5 Centre for MEGA Epidemiology , School of Population Health, The University of Melbourne, Melbourne, Australia; 6 Centre for Health Equity Studies (CHESS), Karolinska Institutet/Stockholm University, Stockholm, Sweden; 7 Clinical Epidemiology, Department of Medicine, Karolinska Institutet, Stockholm, Sweden; 8 Department of Research and Development, Sundsvalls sjukhus, Sundsvall, SE 85186, Sweden

**Keywords:** Asthma, Caesarean section, Child, Epidemiology, Inhaled corticosteroids, Sib pair analysis

## Abstract

**Background:**

Mode of delivery may affect the risk of asthma but the findings have not been consistent and factors shared by siblings may confound the associations in previous studies.

**Methods:**

The association between mode of delivery and dispensed inhaled corticosteroid (ICS) (a marker of asthma) was examined in a register based national cohort (n=199 837). A cohort analysis of all first born children aged 2-5 and 6-9 years was performed. An age-matched sibling-pair analysis was also performed to account for shared genetic and environmental risk factors.

**Results:**

Analyses of first-borns demonstrated that elective caesarean section was associated with an increased risk of dispensed ICS in both 2-5 (adjusted odds ratio (aOR)=1.19, 95% confidence interval (CI) 1.09-1.29) and 6-9 (aOR=1.21, 1.09-1.34) age groups. In the sibling-pair analysis, the increased risk associated with elective caesarean section was confirmed in 2-5 year olds (aOR=1.22, 1.05-1.43) but not in 6-9 year olds (aOR=1.06, 0.78-1.44). Emergency caesarean section and vacuum extraction had some association with dispensed ICS in the analyses of first-borns but these associations were not confirmed in the sibling-pair analyses.

**Conclusions:**

Confounding by familial factors affects the association between mode of delivery and dispensed ICS. Despite this confounding, there was some evidence that elective caesarean section contributed to a modestly increased risk of dispensed ICS but only up to five years of age.

## Introduction

A moderate (20%) increase in asthma prevalence has been described in children delivered by caesarean section [1,2]. A number of studies have also suggested that instrumental vaginal delivery (vacuum extraction and forceps) may contribute to the development of asthma [3-5]. This represents a major public health concern, as there has been a substantial increase in deliveries by caesarean sections and vacuum extractions in high and middle-income countries all over the world [6].

Most previous studies have used a simple dichotomised categorisation of deliveries as being vaginal or caesarean, not taking into account the important differences between elective and emergency caesarean sections, and unaided and assisted vaginal deliveries. It has been hypothesed that the increased risk of asthma in children born by caesarean section is due to a lack of microbial exposure during labour, via the vagina, resulting in delayed and altered colonization of the infant gut [7,8]. Altered microbial colonization may be more pronounced in children born by elective caesarean sections, where rupture of the membranes prior to delivery is rare. Children delivered by elective caesarean section may also be at increased risk of neonatal respiratory morbidity [9] and asthma later in childhood [10], as they do not experience the stress of labour, which helps initiate the infant lungs to switch from secretion to absorption [9,11]. There is also some evidence that instrumental vaginal delivery contributes to an increased risk of asthma [3-5], possibly because of the prolonged maternal stress associated with these deliveries [4]. However, instrumental vaginal deliveries, and emergency caesarean sections, may be markers for complications in pregnancy, and it may be these complications, rather than mode of delivery, that confers the increased risk of asthma.

Some of the inconsistencies in previous studies may be due to insufficient control of confounding, differences in study size, age at outcome assessment and outcome measures.

In this study, we used a Swedish national cohort to overcome some of these obstacles to determine if mode of delivery affects the risk of dispensed ICS, as an indicator of asthma, whilst taking into account relevant socio-demographic and perinatal confounders. The large study population has enabled us to both detect very small differences, and to examine these effects in an aged-matched sibling pair analysis, to account for shared genetic and environmental exposures in the family. A recent and similar Swedish registry-based study did not detect any association between elective caesarean section and asthma in sibling-pairs aged 9 and 12 years. In contrast, association by indication seemed to explain an increased risk for asthma related to emergency caesarean section [12]. The aim of our study was to assess whether elective caesarean section had any association with asthma medication in children born at term and aged less than 9 years and whether the association between mode of delivery and asthma was affected by familial determinants. A potential effect from mode of delivery on the risk of asthma could be transient and only observed in the youngest age groups. We have therefore assessed whether the associations between mode of delivery and asthma medication differed between children aged 2-5 years and 6-9 years.

## Methods

This study was based on information from the national health data bases held by the Swedish National Board of Health and Welfare and Statistics Sweden. Cross-linkage between registers was performed using each individual’s unique personal identification number. This study was approved by the regional ethics committee in Stockholm.

The catchment population for this study were all full term (gestational week 37-<42), singleton children born in Sweden between 1/1/1999 and 31/12/2006, according to the Swedish Medical Birth Register. From this population we selected children who were residents in Sweden on December 2009 (according to the Register of the Total Population), were offspring of two native Swedish parents, had no major malformations reported at birth by the attending paediatrician or cerebral palsy according to the Hospital Discharge Register. Children with improbable combinations of birth weight and gestational age (<-6SD and >3SD) were excluded, as were children with both instrumental vaginal delivery and caesarean section delivery recorded.

Information about parity, maternal age (at childbirth), measured maternal BMI in early pregnancy, mode of delivery, smoking habits in early pregnancy, maternal diseases and pregnancy complications was collected from Swedish Medical Birth Register. From the same register, perinatal data about sex, gestational age, birth weight, and low Apgar scores (<7 at five minutes) of the offspring were collected. Birth weight and gestational age were used to create the indicator of small -for-gestation-age (SGA, <2 SD) and large-for-gestational-age (LGA, >2 SD) [13]. Mode of delivery was categorised into; non-assisted vaginal delivery, emergency caesarean section, elective caesarean section and vacuum extraction. Classification of the caesarean section as emergency or elective is often missing in the register. However, information concerning onset of labour is rarely missing. Therefore, caesarean section was defined as emergency if the operation was made after the onset of labor and otherwise considered as elective. Maternal diseases and pregnancy complications were coded according to the International Classification of Diseases, tenth revisions.

Data on parental education was defined as the highest formal education attained. Educational level was categorised by years of education into <10 years, 10-12 years, 13-14 years and >14 years. Social welfare benefits received were collected from the Income and Enumeration survey for 2008 and dichotomised into any versus none.

We created three proxy indicators for asthma based on data of retrieved prescriptions from the Swedish Prescribed Drug Register, which contains all drugs prescribed and dispensed in Swedish pharmacies, excluding drug treatment in hospital care. The indicators “dispensed inhaled corticosteroid (ICS)” was defined as the purchase of at least one prescription of inhaled steroids, either by itself (Anatomical Therapeutic Chemical [ATC]-code starting with R03BA) or in combination with other agents (ATC codes R03AK06 or R03AK07) while dispensed ICS at least twice was defined as purchase of at least two prescriptions during the same calendar year with these definitions.. Finally, an indicator labeled “any asthma medication“ was defined as the purchase of at least one prescription during a calendar year with an ATC-code that starts with R03 (any medication for obstructive lung disease).

In the cohort of firstborns, ICS during 2009 was used as our primary outcome variable, while dispensed ICS from all four years (2006 to 2009) was used to create age-equalised comparisons for the sibling-pair analysis. Parental ICS was defined as receipt of an ICS during 2009 for all analyses and was used as a proxy for parental asthma.

There were considerable regional differences in the purchase of asthma medications reflecting regional differences in prevalence of asthma as well as differences in access to care and prescription patterns. A four-category *county* variable with different levels of retrieval of prescribed ICS was created to adjust the analysis to these regional differences.

### Statistical analysis

Two related forms of analysis were performed. In the first, all first born children were included, and unconditional logistic regression models were used to assess the relationship between the different modes of delivery and risk of dispensed ICS by the child during 2009. Model 1 was adjusted for year of birth and sex only, in Model 2 we added other socio-demographic confounders (including smoking and maternal age) and parental dispensed ICS, in Model 3 we added perinatal risk factors associated with mode of delivery.

In a second analysis, the effect of mode of delivery was assessed within sibling pairs discordant for mode of delivery and asthma medication, using conditional logistic regression (children grouped according to sharing the same mother). In this analysis we have adjusted for the potential confounders that might vary between the two pregnancies, but not for confounders that vary between mothers only. Model 1 was adjusted for sex, in Model 2 we added maternal age and parity and Model 3 was also adjusted for SGA, LGA, preeclampsia, maternal body mass index, maternal diabetes and gestational diabetes and neonatal respiratory distress, Sibling-pairs were selected on the basis of having the widest age gap possible, whilst still being able to match siblings on age. In this analysis sib age was equalised by taking ICS dispensing at the age of the youngest sib in the analysis in 2009 from the calendar year 2006 to 2008.

For all forms of analysis, potential non-linear effects of maternal BMI were assessed using the “fracpoly” command within Stata (release 10.1, College Station, Texas). Effect modification, by gender, parity, maternal smoking, parental asthma (dispensed ICS) was assessed using Wald tests. As the nature of wheeze changes with age, the exposure to asthma medications was classified into two discrete age groups, 2-5 and 6-9 years of age.

## Results

Altogether 13 179 (12.7%) boys and 9934 (9.2%) girls in the age two to nine years had purchased any asthma medication in 2009, and of these 7180 (6.9%) boys and 4722 (4.6%) girls had retrieved at least one prescription of ICS.

Dispensed asthma medication increased gradually with increasing maternal BMI (Table 1). Children whose mother had a pre-existing condition or a pregnancy complication were more likely to receive asthma medication. In addition, being born in gestational week 37-38 had higher rates of medication than those born in week 39-41. Having had neonatal respiratory problems was also associated with a higher risk of asthma medication

**Table 1:** Asthma medication by socio-demographic and neonatal indicators (%) in 2-9 year olds

**N=199 837**					
			**Any ICS**	**ICS twice**	**Any asthma medication**
		**N**	**%**	**%**	**%**
Sex	Boys	100 688	7.1	3.6	10.9
	Girls	99 149	4.6	2.4	7.6
Age (in years)	2-3	53 346	7.1	3.9	12.8
	4-5	52 411	5.8	3.0	9.0
	6-7	49 214	5.2	2.5	7.5
	8-9	44 866	5.2	2.5	7.5
Maternal age at delivery (years)	−24	43 079	5.6	2.6	9.1
	25-34	138 135	5.9	9.3	9.3
	>34	18 623	6.2	10.0	10.0
Maternal body mass index	Underweight	4311	5.1	2.3	8.4
	Normal	117 552	5.5	2.9	8.8
	Overweight	38 443	6.4	3.4	10.1
	Obese 1	10 955	7.1	4.0	11.0
	Obese2	4104	7.9	3.1	12.4
	Missing	24 472	5.9	3.0	9.4
Gestational age (weeks)	39-41	159 368	5.7	2.9	9.0
	37-38	40 469	6.7	3.5	10.3
Hypertension	Preeclampsia	2457	7.5	3.7	11.5
	Pregestational	7409	6.4	3.5	9.8
Diabetes type 1	Yes	708	5.6	3.5	9.6
Gestational diabetes	Yes	1163	7.6	4.4	11.4
Oligohydramniom	Yes	2094	6.8	3.6	11.0
Premature ruptures ruptures of membranes	Yes	750	5.9	2.9	9.5
Low apgar score	Yes	1980	6.7	3.5	9.6
Small for gestational age (SGA)	Yes	4388	7.1	3.9	11.2
Large for gestational age (LGA)	Yes	3840	6.7	3.6	10.2
Any neonatal respiratory complication	Yes	4237	6.6	3.7	10.4
Maternal smoking	10+	3508	6.3	3.0	10.2
	1-9	13 693	5.9	2.8	9.8
	0	169 700	5.8	3.0	9.2
	Missing data	12 936	6.6	3.4	10.2
Maternal asthma medication	Yes	5916	15.2	8.8	20.4
Paternal asthma medication	Yes	5491	11.1	6.3	15.9
Maternal education (years)	<=9	12 461	5.9	2.7	9.7
	10-12	86 807	5.9	2.9	9.5
	13-14	28355	5.9	3.0	9.2
	15+	71 808	5.8	3.2	9.0
County category	1	48 002	6.4	3.2	10.4
	2	36 950	6.0	3.1	9.3
	3	76 915	5.6	3.1	8.9
	4	37 970	5.5	2.7	8.6
*All*		199 837	5.9	3.0	9.3

Table 2 shows that mothers giving birth by caesarean section or vacuum extraction were more likely than those born by non-assisted vaginal delivery to be older and to have chronic disorders, pregnancy complications and asthma medications. Maternal overweight or obesity was more common among children delivered by caesarean section but not among those born by vacuum extraction deliveries. Furthermore, newborns delivered instrumentally (both caesarean section and vacuum extraction) more often had respiratory distress, low Apgar scores, meconium aspiration and were classified as SGA or LGA. Some maternal complications, *e.g.* hypertension and diabetes were more common in those who were delivered by elective caesarean section compared with those who were delivered by emergency caesarean section. In contrast, low Apgar was overrepresented after emergency caesarean section and vacuum extraction.

**Table 2:** Asthma medication, socio-demographic and neonatal indicators by mode of delivery in 2-9 year olds

**N=199 837**					
		**Vaginal**	**Elective CS**	**Emergency CS**	**Vacuum extraction**
		**N=143 347**	**N=16 050**	**N=13 875**	**N=26 565**
		**%**	**%**	**%**	**%**
Any asthma medication (child)	Yes	8.9	11.3	10.2	9.8
ICS (child)	Yes	5.6	7.1	6.5	6.4
ICS at least twice (child)	Yes	2.8	3.8	3.6	3.4
ICS (mother)	Yes	2.7	4.2	3.5	3.1
ICS (father)	Yes	2.6	3.7	3.0	2.8
Maternal age at delivery (years)	11-24	24.1	13.6	14.4	16.3
	25-34	68.5	67.1	71.6	72.3
	>34	7.4	19.3	14.0	11.4
Maternal body mass index	Underweight	2.3	1.8	1.3	2.0
	Normal	60.5	52.4	49.7	58.5
	Overweight	18.4	21.5	23.7	20.1
	Obese1	5.0	7.6	8.4	5.2
	Obese2	1.8	3.6	3.5	1.9
	Missing	12.0	13.1	13.4	12.3
Gestational age					
(weeks)	37-38	18.1	53.8	16.7	13.3
Hypertension	Preeclampsia	1.1	2.1	1.2	1.3
	Pregestational	3.0	11.2	2.7	3.6
Diabetes type 1	Yes	0.2	1.6	0.6	0.5
Gestational diabetes	Yes	0.5	1.3	0.7	0.5
Low Apgar score	Yes	0.5	1.3	2.4	2.6
SGA	Yes	1.8	4.6	3.2	2.2
LGA	Yes	1.4	4.1	4.2	2.2
Any neonatal respiratory complication	Yes	1.6	3.1	3.9	3.3
Maternal smoking	Yes	8.7	9.4	8.6	7.4
Social welfare	Yes	2.5	2.0	1.8	1.6
Maternal education	<10 years	6.6	6.2	5.5	4.9
	10-12	43.4	44.0	44.6	43.2
	13-14	14.0	15.1	15.2	14.5
	>14	36.0	34.6	34.7	37.3
County category	1	22.6	29.2	26.6	27.2
	2	19.2	17.2	17.8	15.8
	3	39.4	35.5	36.8	36.2
	4	18.8	18.1	18.8	20.9

Abbreviations: CS, caesarean section; ICS, inhaled corticosteroid use; LGA, large for gestational age; SGA, small for gestational age.

**Figure 1:**
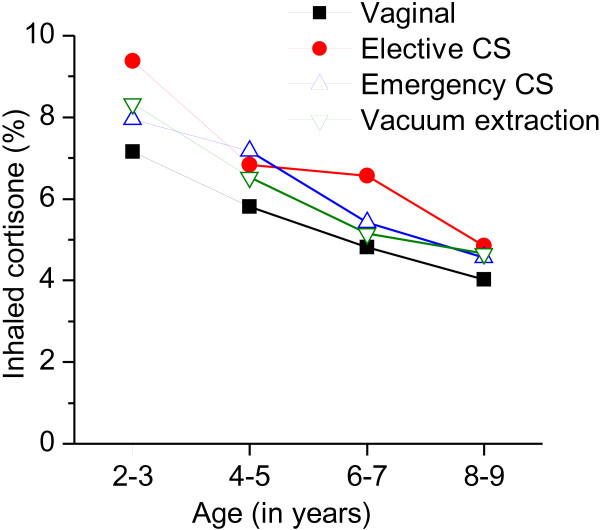
Prevalence of dispensed inhaled corticosteroids at least once in different age groups in relation to mode of delivery.

Children delivered by non-instrumental vaginal delivery had the lowest rates of asthma medication in all age groups (Figure 1). Children delivered by elective caesarean section had the highest rates in all age groups except the 4-5 year olds while emergency caesarean section and vacuum extraction had a similar rate in all age groups.

When compared to vaginal delivery without assistance, both elective and emergency caesarean sections were associated with a modest increase in risk of any dispensed ICS (at least one prescription) in the 2-5 year olds after adjustment for socio-demographic confounders (Table 3, model 2),. Adjusting for perinatal risk factors attenuated these risks slightly (model 3). In the 6-9 year olds, children delivered by elective caesarean section had an increased risk (adjusted odds ratios (aOR) 1.21, 95% confidence interval (CI) 1.09-1.34 for model 3). The association with emergency caesarean section delivery and dispensed ICS was weaker, and no evidence of an association remained after adjustments for the confounding and mediating variables (aOR 1.05, 95% CI 0.93-1.17 for model 3).

**Table 3:** **The association between mode of delivery and dispense of at least one prescription of inhaled cortisone**^**1**^

**Mode of delivery**	**Inhaled corticosteroid use**			**Unadjusted**		**Model 1**		**Model 2**		**Model 3**	
	**No of cases**	**Total No**	**%**	**OR**	**95% CI**	**aOR**	**95% CI**	**aOR**	**95% CI**	**aOR**	**95% CI**
**2-5 years**											
Vaginal^2^	4597	74694	6.2	1.0	1.0	1.0	1.0	1.0	1.0	1.0	1.0
Elective CS	698	9061	7.7	1.27	1.17, 1,38	1.26	1.16, 1,37	1.22	1.12, 1,33	1.19	1.09, 1,29
Emergency CS	546	7478	7.3	1.20	1.10, 1.32	1.18	1.08, 1.29	1.17	1.06, 1.28	1.14	1.04, 1.25
Vacuum extraction	981	14524	6.8	1.10	1.03, 1,19	1.08	1.00, 1,16	1.07	1.00, 1,15	1.07	0.99, 1,15
**6-9 years**											
Vaginal^2^	3391	68653	4.9	1.0	1.0	1.0	1.0	1.0	1.0	1.0	1.0
Elective CS	442	6989	6.3	1.30	1.17, 1.44	1.29	1.17, 1.43	1.24	1.12, 1.38	1.21	1.09, 1.34
Emergency CS	358	6397	5.6	1.14	1.02, 1.28	1.11	1.00, 1.25	1.08	0.78, 1.21	1.05	0.93, 1.17
Vacuum extraction	711	12041	5.9	1.21	1.01, 1,31	1.17	1.07, 1.27	1.16	1.06, 1.26	1.15	1.05, 1.25

Crude and adjusted odds ratios after non-conditional logistic regression.

Abbreviations: CI, confidence interval; aOR, adjusted odds ratio; OR, odds ratio.

**Model 1** is adjusted for year of birth and sex. **Model 2**: also adding: maternal and paternal asthma medication. socioeconomic indicators (maternal education. social welfare) maternal age, maternal smoking, urban/rural living, county. **Model 3** also adding factors that may increase the risk of caesarean section: maternal history of diabetes and hypertension, premature rupture of the membranes, preeclampsia, gestational diabetes, gestational hypertension, maternal body mass index, small for gestational age, large for gestational age, maternal fever during labour,. chorioamnionitis, meconium aspiration, neonatal respiratory distress, transient tachypnoea.

^1^Only first born children included, ^2^Non-instrumental vaginal delivery.

Delivery by vacuum extraction was associated with a slightly increased risk of dispensed ICS, in the 6-9-years olds (aOR 1.15, 95% CI 1.05-1.25), after adjustment for confounders and a borderline increased risk in the 2-5 year olds (aOR 1.07, 95% CI 0.99-1.15). Interaction analyses showed that the effect of mode of delivery was similar in boys and girls.

We have also assessed the associations between mode of delivery and at least two dispensed prescriptions of ICS as the outcome. The findings differed only slightly from the analyses based on at least one dispensed prescription of ICS (Additional file 1: Table S1)

### Sibling pair analysis

In the sibling pair analysis, of the 30 515 sibling pairs aged 6-9 years, 2290 were discordant for ICS use, and of these 569 were also discordant for mode of delivery (Additional file 2: Table S2). In this age group, all effects were marginal with wide confidence intervals when compared to the unconditional analysis (Table 4). Of the 80 140 sib pairs aged 2-5 years, 1 965 were discordant for both ICS use and mode of delivery. In this age group children delivered by elective caesarean section had an increased risk of asthma medication use, when compared to their age matched sibling (crude OR 1.21, 95% CI 1.05-1.41 and aOR 1.19, 95% CI 1.09-1.29).

**Table 4:** Conditional logistic regression models for the association between mode of delivery and dispense of at least one prescription of inhaled cortisone at least once in discordant sibling-pairs

**Mode of delivery**	**Unadjusted**		**Model 1**		**Model 2**		**Model 3**	
	**OR**	**95% CI**	**aOR**	**95% CI**	**aOR**	**95% CI**	**aOR**	**95% CI**
**2-5 years** (n= 7 688 contrasting sib pairs)								
Vaginal^1^	1.0	1.0	1.0	1.0	1.0	1.0	1.0	1.0
Elective CS	1.21	1.05, 1,41	1.19	1.03, 1.39	1.22	1.05, 1.42	1.23	1.05, 1.43
Emergency CS	0.88	0.74, 1.06	0.84	0.70, 1.02	0.95	0.79, 1.15	0.95	0.78, 1.14
Vacuum extraction	0.94	0.83, 1.06	0.89	0.79, 1.01	1.06	0.92, 1.21	1.05	0.92, 1.20
**6-9 years** (n=2 290 contrasting sib pairs)								
Vaginal^1^	1.0	1.0	1.0	1.0	1.0	1.0	1.0	1.0
Elective CS	1.05	0.79, 1.40	1.08	0.80, 1.46	1.09	0.81, 1.47	1.06	0.78, 1.44
Emergency CS	1.05	0.75, 1.46	1.00	0.71, 1.42	1.03	0.73, 1.46	1.02	0.72, 1.44
Vacuum extraction	0.93	0.74, 1.17	0.89	0.70, 1.12	0.91	0.71, 1.17	0.92	0.72, 1.18

Odds ratios in relation to the reference, Non-instrumental vaginal delivery in different age groups.

Abbreviations: CI, confidence interval; aOR, adjusted odds ratio; OR, odds ratio.

**Model 1** – adjusted for infant gender, **Model 2** – also adjusted for maternal age and parity, **Model 3** – also adjusted for small for gestational age, large for gestational age, preeclampsia, maternal body mass index, maternal diabetes and gestational diabetes and neonatal respiratory distress.

^1^Non-instrumental vaginal delivery.

## Discussion

Our analyses of first-borns demonstrated that elective caesarean sections were associated with modestly increased risk of dispensed ICS, a marker of asthma. The sibling-pair analysis, which inherently adjusts for shared environmental and genetic factors, confirmed this association in elective caesarean in the two to five year olds, but not in the older children. The associations between emergency caesarean section and vacuum extraction and risk of dispensed ICS observed in the analyses of first-borns where not confirmed in the sibling-pair analyses, suggesting these effects are due to residual confounding.

Several large cohort studies from Scandinavian countries have suggested that emergency as well as elective section could affect the risk of asthma [5,14] or asthma hospitalisation in young children [3]. The conventional, unconditional logistic regression, analysis in our study indicated that emergency caesarean sections and vacuum extraction were associated with an increased risk of asthma medication but these associations disappeared completely in the sibling pair analyses suggesting that these effects in the unconditional logistics regression were due to residual confounding by familial risk factors. It remains unclear what these unmeasured confounding factors in the conventional analysis could be. We have adjusted for a range of potential confounders, including socio-economic status, parental asthma, and pregnancy and neonatal complications. Adjustment for these factors did not greatly alter the observed associations. Maternal anxiety is associated with an increased risk of pregnancy complications [15] and emergency caesarean sections [16] and maternal stress during pregnancy could contribute to an increased risk of childhood asthma [17]. Although we did adjust our analysis for proxy indicators for parental asthma (dispensed ICS), there may well be residual genetic confounding that connects perinatal complications with asthma later in life.

The current study underlines the difficulties to control for confounding in conventional analyses of mode of delivery and asthma. It is based on register data covering the whole Swedish population. There is no recall or selection bias. However, some of the indicators in the registers are crude proxies for the true exposures [18]. It is particulary difficult to cover life style factors, and the specific indications for elective caesareans. These factors may affect mode of delivery [19,20] as well as the risk of asthma [21], use of medication and access to health care [22].

Studies with a sibling design are a powerful tool to control for familial confounding due to genetic or environmental factors [23]. With the exception of the recent study by Almqvist *et al*[12], this design has not been used in epidemiological studies of mode of delivery and asthma. A prerequisite is a sufficient number of discordant sibling pairs. Our study is based on all ethnic Swedish children born term between 1992 and 2008 with data available on over 110 000 sibling pairs. Despite these quite impressive sibling populations, only 1965 sibling pairs at age 2-5 and 569 at age 6-9 were discordant for both mode of delivery and asthma medication use, and thus were informative for this analysis. These sample sizes were insufficient to obtain precise estimates of these comparatively small effects, thus requiring cautious interpretation. In comparison, Almqvist *et al.* had 1005 informative sibling pairs available in their study. Although the majority of our findings are consistent with those of Almqvist *et al.* they observed an association between emergency caesarean section and increased risk of asthma [12], while we did not. This may be due to multiple differences in the design of these two studies. Specifically, we excluded all children born pre-term, and those who had malformations or cerebral palsy. These conditions may result in delivery by emergency caesearan section and increased risk of developing various respiratory illnesses including asthma.

Childhood asthma is a heterogenous disease and a number of phenotypes with partly different risk factors have been identified [24]. Almqvist *et al* did not observe any association between elective caesarean section and an increased risk of asthma in sibling-pairs aged 9 and 12 years [12]. Our study suggests that elective caesarean section could be associated with a slightly increased risk of transient wheeze up to five years of age. Maternal complications during pregnancy have previously been linked to an increased risk of childhood wheeze [25]. Hypertension and diabetes were more likely in women who were delivered by elective caesarean section but the increased risk for asthma medication persisted also after adjustment for these factors. However, we cannot exclude that other undetected complications contributed to the increased risk of asthma medication in preschoolers.

This study has a number of limitations. Defining asthma within epidemiological studies is a challenge. We have chosen to use prescription of inhaled corticosteroids (ICS) to define current symptoms of asthma. We have previously used a similar definition [26] as has a similar study from Finland [5] A number of studies have assessed the validity of prescription registry based information on asthma medicine use against parent report of asthma [27], standardised questionnaire based definitions of asthma [28], and doctor diagnosis [29] in similar settings to the current study. These studies generally find that prescription registry data provides a reasonably valid definition of asthma. We have elected to exclude use of beta-agonists from our primary definition of the outcome, as these are a less specific marker for asthma.

However, dispensed medication as a proxy for asthma is affected by a number of factors at different levels such as awareness of the symptoms by the child and the parents, severity of asthma, health seeking behavior, diagnostic criteria by the doctor, attitude to medication and its related costs. If one sibling has asthma, other siblings are more likely to be diagnosed and get a prescription for an asthma medication than children in families where the symptoms of asthma are less familiar. Sharing of medication may occur in families where several members suffer from asthma. When asthma is diagnosed compliance with medication probably differ in different families, and this will have an effect on subsequent prescriptions. Many of these family effects could contribute to a negative outcome and some of these such as sharing of medication could weaken the associations also in the sibling analyses. Using purchase of ICS may imply that we have missed individuals (parents or children) with undiagnosed or mild asthma. Therefore, residual confounding may explain some of the reduced effect sizes in the sibling analysis.

In young children, dispensed ICS is a proxy for respiratory illness, but not always for asthma. as diagnosis of asthma is very difficult in preschool children. By the age of 6 years, the prevalence of transient viral wheeze has largely passed, and a diagnosis of asthma is much easier to confirm, making this a reasonable time point to divide the cohort of children. Variability in prescription pattern could dilute the association between asthma medication and mode of delivery in young children [30]. For this reason, we have also excluded children aged less than two years, and used ICS rather than all asthma medication as our main outcome. Children with nonspecific clinical symptoms sometimes get one prescription of ICS as a test for asthma. One may therefore ague that at least two prescriptions of ICS would have been a better proxy for asthma in our study but we have tested analyses based on at least two prescriptions of ICS and the findings were fairly similar.

An association between caesarean section and asthma could also be weakened or even missed, if potential effects are restricted to specific genetic variants or subgroups of asthma. We do not have direct measures for atopy in this study. Therefore, we cannot exclude that mode of delivery could affect allergic induced symptoms. Moreover, we have no information in the registers concerning postnatal exposures. However, a recent birth cohort study failed to identify modification of the association of between mode of delivery and childhood asthma by a range of key postnatal exposures, including breast-feeding behavior and age at day care entry [14].

We cannot exclude the risk of some misclassification of the caesarean sections. “Elective” caesarean sections were not necessarily optional. We considered a caesarean section to be emergent if the operation was made after the onset of labour. Therefore, caesarean sections due to emergencies such as fetal distress or a severe maternal complication were defined as elective if the caesarean sections were performed without preceding labour. Although this would have resulted in some misclassification between emergency and elective caesarean sections, as this reason for cesarean section is relatively uncommon, we consider it to be an unlikely source of a major bias in this study. The major indications in Sweden for elective caesarean sections after 36 weeks are breech presentation, previous caesarean section, cephalopelvic disproportion and psychosocial factors. They accounted for more than 80% of all elective caesarean sections between 1996 and 2007 [31].

To conclude, elective caesarean section contributes to a modestly increased risk of asthma medication, but only up to five years of age. The associations between emergency caesarean section or vacuum extraction and asthma medication seen in the firstborns appear to be due to residual confounding by familial factors.

## Competing interests

All authors declare that they have no competing interests.

## Authors’ contributions

LB contributed to the design of the study, conducted the literature review, prepared the first draft and revised the final version of the manuscript. CE contributed to the design of the study, conducted the data analyses and revised the final version of the manuscript. AL designed the study’s analytic strategy, performed the sibling analyses and revised the manuscript. AH designed the study and the analytic strategy, performed register linkages, created the dataset, performed some statistical analyses, directed the implementation including quality assurance and control and contributed to all parts of the manuscript. All authors read and approved the final manuscript.

## Supplementary Material

Additional file 1: Table S1 The association between mode of delivery and use of inhaled cortisone at least twice during 2009. Crude and adjusted odds ratios after non-conditional logistic regression. Click here for file

Additional file 2: Table S2 Association between mode of delivery and inhaled cortisone at least once in discordant sib-pairs. Click here for file
