# Supplementary material for: Confounding with familial determinants affects the association between mode of delivery and childhood asthma medication – a national cohort study
Source: Allergy Asthma Clin Immunol. 2013 Apr 16;9(1):14. doi: 10.1186/1710-1492-9-14 (PMC3643829; doi:10.1186/1710-1492-9-14)
Supplement: Additional file 1: Table S1 — The association between mode of delivery and use of inhaled cortisone at least twice during 2009. Crude and adjusted odds ratios after non-conditional logistic regression. [file 1710-1492-9-14-S1.docx]

**Supplemental Table 1**. The Association Between Mode of Delivery and Use of Inhaled Cortisone at least twice during 2009. Crude and Adjusted Odds Ratios After Non-conditional Logistic Regression

| **Mode of delivery** | **Inhaled corticosteroid use** | | | **Unadjusted** | | **Model 1** | | **Model 2** | | **Model 3** | |
| --- | --- | --- | --- | --- | --- | --- | --- | --- | --- | --- | --- |
|  | **No of cases** | **Total No** | **%** | **OR** | **95% CI** | **aOR** | **95% CI** | **aOR** | **95% CI** | **aOR** | **95% CI** |
| **2-5 years** |  |  |  |  |  |  |  |  |  |  |  |
| Vaginal^1^  Elective CS  Emergency CS  Vacuum extraction | 2 416  394  320  559 | 74 694  9 061  7 478  14 524 | 3.2  4.3  4.3  3.8 | 1  1.36  1.34  1.20 | 1  1.22, 1,52  1.19, 1.51  1.09, 1,32 | 1  1.34  1.32  1.17 | 1  1.21, 1,50  1.17, 1.29  1.07, 1,29 | 1  1.27  1.28  1.15 | 1  1.14, 1,42  1.14, 1.45  1.05, 1.26 | 1  1.24  1.28  1.15 | 1  1.11, 1,41  1.13, 1.44  1.04, 1.26 |
| **6-9years** |  |  |  |  |  |  |  |  |  |  |  |
| Vaginal^1^  Elective CS  Emergency CS  Vacuum extraction | 1 638  211  177  340 | 68 653  6 989  6 397  12 041 | 2.4  3.0  2.8  2.8 | 1.0  1.27  1.16  1.19 | 1.0  1.11, 1.47  1.00, 1.36  1.06, 1,34 | 1.0  1.27  1.14  1.15 | 1.0  1.10, 1.47  0.97, 1.33  1.02, 1.29 | 1.0  1.20  1.09  1.13 | 1.0  1.03, 1.39  0.93, 1.28  1.01, 1.28 | 1.0  1.16  1.08  1.13 | 1.0  1.00, 1.35  0.92, 1.27  1.00, 1.27 |

Abbreviations: CI, confidence interval; aOR, adjusted odds ratio; OR, odds ratio

**Model 1** is adjusted for year of birth and **sex . Model 2**: also adding: maternal and paternal asthma medication. socioeconomic indicators (maternal education. social welfare) maternal age, maternal smoking, urban/rural living, county. **Model 3** also adding factors that may increase the risk of caesarean section: maternal history of diabetes and hypertension, premature rupture of the membranes, preeclampsia, gestational diabetes, gestational hypertension, maternal body mass index, small for gestational age, large for gestational age, maternal fever during labour,. chorioamnionitis, meconium aspiration, neonatal respiratory distress, transient tachypnoea.

^1^Non-instrumental vaginal delivery
