# Supplementary material for: Confounding with familial determinants affects the association between mode of delivery and childhood asthma medication – a national cohort study
Source: Allergy Asthma Clin Immunol. 2013 Apr 16;9(1):14. doi: 10.1186/1710-1492-9-14 (PMC3643829; doi:10.1186/1710-1492-9-14)
Supplement: Additional file 2: Table S2 — Association between mode of delivery and inhaled cortisone at least once in discordant sib-pairs. [file 1710-1492-9-14-S2.doc]

**Supplementary table 2. Association Between** Mode of Delivery and Inhaled Cortisone at Least Once in Discordant Sib-pairs.

| **Children aged 2-5 years discordant on use of ICS (n=7,688)** | | | | | |  | |
| --- | --- | --- | --- | --- | --- | --- | --- |
|  |  |  | Mode of delivery for the sib who did not use ICS | | | | |
|  |  | Vaginal | Elective CS | Emergency CS | Vacuum extraction | | Total |
| Mode of delivery for the sib who did use ICS | Vaginal | 67.5%  (5193) | 2.6%  (198) | 1.6%  (126) | 5.5%  (424) | | 77.3%  (5941) |
|  | Elective CS | 3.1%  (242) | 5.1%  (391) | 1.9%  (143) | 1.1%  (84) | | 11.2%  (860) |
|  | Emergency CS | 1.5%  (117) | 1.2%  (96) | 1.1%  (84) | 0.4%  (34) | | 4.3%  (331) |
|  | Vacuum extraction | 5.1%  (391) | 1.0%  (75) | 0.5%  (35) | 0.7%  (55) | | 7.2%  (556) |
|  | Total | 77.3%  (5943) | 9.9%  (760) | 5.0%  (388) | 7.8%  (597) | | 100%  (7688) |
| **Children aged 6-9 years discordant on use of ICS (n=2,290)** | | | | | |  | |
|  |  |  | Mode of delivery for the sib who did not use ICS | | | | |
|  |  | Vaginal | Elective CS | Emergency CS | Vacuum extraction | | Total |
| Mode of delivery for the sib who did use ICS | Vaginal | 69.4%  (1589) | 2.2%  (50) | 1.7%  (39) | 5.8%  (133) | | 79.1%  (1811) |
|  | Elective CS | 2.5%  (58) | 3.7%  (85) | 1.5%  (34) | 1.0%  (22) | | 8.7%  (199) |
|  | Emergency CS | 1.6%  (37) | 1.7%  (39) | 1.6%  (36) | 0.3%  (8) | | 5.2%  (120) |
|  | Vacuum extraction | 5.3%  (121) | 0.8%  (19) | 0.4%  (9) | 0.5%  (11) | | 7.0%  (160) |
|  | Total | 78.8%  (1805) | 8.4%  (193) | 5.2%  (118) | 7.6%  (174) | | 100%  (2290) |
